# Post-operative Rehabilitation in Klatskin Tumor: A Rare Case Report

**DOI:** 10.7759/cureus.30315

**Published:** 2022-10-14

**Authors:** Danish Siddiqui, Rebecca Ferreira, Sabih N Khan, Nikita Narwade, Shrikant Mhase, Aishwarya A Pashine, Akshay M Nimje, Roshan Umate

**Affiliations:** 1 Department of Rehabilitation, Mahatma Gandhi Mission (MGM) School of Physiotherapy, Aurangabad, IND; 2 Department of Cardiorespiratory Physiotherapy, Mahatma Gandhi Mission (MGM) School of Physiotherapy, Aurangabad, IND; 3 Department of Community Physiotherapy, Mahatma Gandhi Mission (MGM) School of Physiotherapy, Aurangabad, IND; 4 Department of Research, Narendra Kumar Prasadrao (NKP) Salve Institute of Medical Sciences and Research Centre, Nagpur, IND; 5 Department of Research and Development, Jawaharlal Nehru Medical College, Datta Meghe Institute of Medical Science, Wardha, IND

**Keywords:** case report, peak expiratory flow rate, respiratory physiotherapy, post-operative pulmonary complications, klatskin tumor

## Abstract

Cholangiocarcinoma (CCA), commonly referred to as Klatskin tumor (KT), is a rare cancer that develops from the epithelium of the intra- or extrahepatic bile duct. This case outlines the impact of physiotherapy rehabilitation in a post-operative case of a KT in a 58-year-old male who presented with complaints of abdominal pain, nausea, constipation, and difficulty in urinating and reportedly exhibited generalized weakness, weight loss, and dyspnea. Following investigations such as computed tomography (CT) scan, the patient was diagnosed with a KT for which he underwent hepaticojejunostomy and was kept under observation, following which supervised physiotherapy intervention (PI) commenced from post-operative day (POD) 3. The outcome measure was peak expiratory flow rate (PEFR), whereas the intervention involved diaphragmatic breathing exercises (DBEs), thoracic expansion exercises (TEEs), incentive spirometry (IS), range of motion (ROM) exercises, active cycle of breathing technique (ACBT), and ambulation. After two weeks of treatment, there were an improvement in cough frequency and an appreciable change in vital capacity (VC), and a significant increase in PEFR values was observed.

## Introduction

Klatskin tumor (KT), also known as perihilar or hilar cholangiocarcinomas (CCAs), was initially identified by Klatskin in 1975 on a series of 13 individuals, developing from intra- or extrahepatic bile duct epithelium, and accounts for 3% of all gastrointestinal tumors [1,2]. Additionally, 40% of CCAs are distal extrahepatic, 40% are perihilar or hilar, and only 10% or less are intrahepatic [2]. There are one to two instances per 100,000 people in Western countries and 96 cases per 100,000 people in Northeast Asia. It makes for 1.3%-2.6% of all cancer-related fatalities worldwide each year [3-5].

KT usually shows signs of biliary obstruction with jaundice and pale stools [6]. Other signs and symptoms include fatigue, unintentional weight loss, hepatomegaly, lymphadenopathy, venous thrombosis, abdominal pain, general sensation of illness, dyspnea, and generalized weakness [7,8].

Post-operative pulmonary complications (PPCs) affect 2%-40% of patients and are linked to longer hospital stays and higher rates of morbidity and mortality [9]. The most frequently discussed PPCs in the scientific literature are pneumonia, post-operative fever, respiratory failure, prolonged mechanical ventilation, pleural effusions, pneumothorax, and pulmonary edema [10]. The application of chest physiotherapy in a post-operative case of KT and the evaluation of the outcomes were the goals of this case report.

## Case presentation

A 58-year-old male presented with complaints of abdominal pain, nausea, constipation, and difficulty in urinating, all of which led to widespread weakness, weight loss, and dyspnea. On examination and investigations that involved computed tomography (CT) and ultrasonography of the abdomen and pelvis, the frozen section report showed the proximal margin of the cystic bile duct (CBD) involved by the tumor with the distal margin of CBD free of tumor, and the histopathology report showed a distal margin of CBD negative for malignancy, whereas the proximal margin of CBD showed occasional dysplastic cells. Additionally, all vital parameters were noted such as heart rate (83 beats per minute), respiratory rate (19 breaths per minute), peripheral capillary oxygen saturation (SpO_2_ = 95%), and peak expiratory flow rate (PEFR = 150 L/minute). Based on these clinical findings and diagnostic assessment, the patient was diagnosed with KT, for which he underwent hepaticojejunostomy. After surgical intervention and before initiating physiotherapy intervention (PI), the main diagnostic assessment involved was PEFR, which is a measure of the highest expiratory flow following maximal inspiration and was measured with a handheld peak flow meter, which was found to be 150 L/minute prior to treatment.

**Figure 1:**
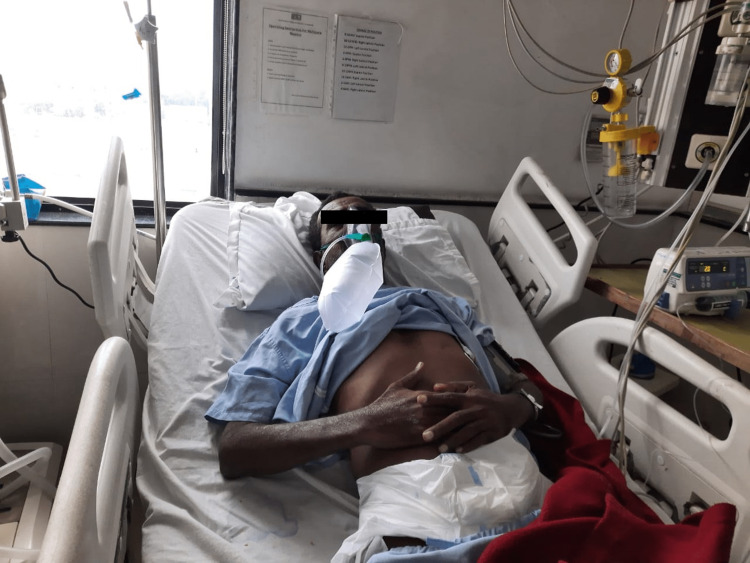
Diaphragmatic Breathing Exercise.

Before commencing PI, the purpose of this case report was explained to the patient, and informed consent was received before planning the therapeutic intervention, following which supervised PI commenced from post-operative day (POD) 3. Physiotherapy regimen was performed for 25 minutes a day, six days a week, for two consecutive weeks. POD 3 and 4 involved positioning from supine to supported sitting to prevent complications such as bed sores, diaphragmatic breathing exercises (DBEs) (Figure 1), and incentive spirometry (IS) with one set of three to five repetitions to facilitate controlled breathing and slow deep breathing along with visual feedback and monitoring inspiratory effort, respectively. Further, POD 5-8 included positioning from unsupported sitting to standing, DBEs and IS with two sets of five repetitions (twice in a day), and thoracic expansion exercises (TEEs) with one set of five repetitions with three-second end-inspiratory breath hold to facilitate collateral channel ventilation. Additionally, POD 9-12 involved positioning in standing, DBEs and IS with two sets of five repetitions (thrice in a day) with three-second hold in IS, TEEs with two sets of five repetitions with three-second end-inspiratory hold, and three to five cycles of ACBT to facilitate airway clearance by loosening, collecting, and evacuating secretions, following which the patient was discharged.

**Figure 2:**
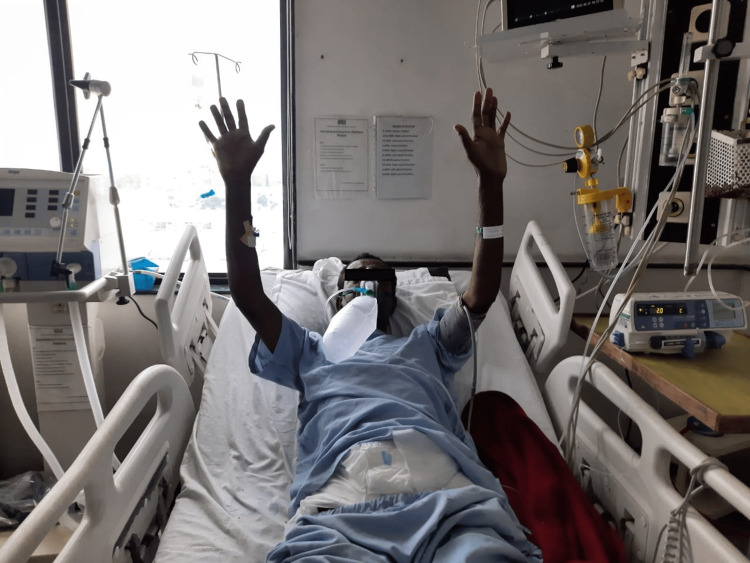
Upper Limb ROM Exercise. ROM = Range of Motion

**Figure 3:**
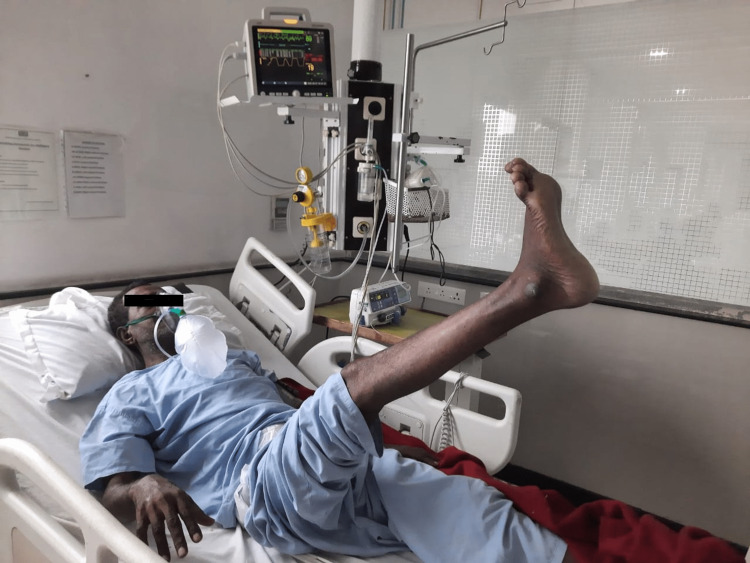
Lower Limb ROM Exercise. ROM = Range of Motion

To prevent circulatory and musculoskeletal complications, additional exercises performed once a day were ankle-toe movements, heel slides with five sets of 10 repetitions, and active range of motion (ROM) exercises for bilateral upper (Figure 2) and lower (Figure 3) limbs with two sets of 10 repetitions each. Aerobic exercises to improve functional capacity such as spot marching and ambulation were also inculcated in the PI.

Follow-up and outcomes demonstrated that after two weeks of targeted respiratory PI, it was observed that there was an increase in the patient's functional capacity, reduced levels of dyspnea, improved sputum production, and a significant increase in the post-PEFR values (Table 1).

**Table 1:** Parameters Describing Pre-treatment and Post-treatment Values. SpO_2_ = Oxygen Saturation; PEFR = Peak Expiratory Flow Rate

Parameters	Pre-treatment values	Post-treatment values
SpO_2_	95%	100%
Heart rate	83 breaths per minute (bpm)	92 bpm
Respiratory rate	19 breaths per minute	23 breaths per minute
PEFR	150 L per minute	210 L per minute

## Discussion

This case report highlights the importance of physiotherapy rehabilitation in a post-operative case of KT, as after surgical intervention, PPCs are a significant contributor to anesthesia- and surgery-related morbidity and mortality, and they also prolong hospital stays [11]. Functional residual capacity (FRC) is typically reduced by at least 0.5 L following abdominal surgery. Low FRC increases the chances of atelectasis and PPCs, as well as other detrimental impacts on lung volumes such as pain and immobility [12].

Lung expansion interventions, such as chest physiotherapy exercises, deep breathing exercises, and continuous positive airway pressure, along with other medical interventions, can be effectively used to supervise PPCs associated with post-operative cases of abdominal surgery. Fundamental measures such as positioning and ambulation are probably best for reducing incidences of PPCs. Previous studies back our case report that inferred, post-treatment PEFR along with other vitals displayed significant improvement [11,13].

PEFR was considered as an outcome measure as this meter is easy to use and portable. Precedent studies have examined correlations between PEFR and other measures of pulmonary function, and additionally, they discovered a high correlation between reduced PEFR readings and respiratory symptoms, as well as between PEFR with functional capacity and regular physical activity [14]. Hence, accurate physiotherapeutic interventions lead to PPC reduction, adding quality-adjusted life years and a stronger signal toward reducing downstream hospital costs.

## Conclusions

This case report emphasizes the efficacy of supervised respiratory physiotherapy in a patient with a post-operative case of Klatskin tumor. Post-operative pulmonary complications after surgical interventions are usual and delay the process of healing. There is no substantial evidence of such a case previously. Hence, physiotherapeutic intervention showed significant improvement in vital capacity and post-peak expiratory flow rate values, thus reducing post-operative pulmonary complications.
